# Rewards of Compassion: Dispositional Compassion Predicts Lower Job Strain and Effort-Reward Imbalance Over a 11-Year Follow-Up

**DOI:** 10.3389/fpsyg.2021.730188

**Published:** 2021-09-28

**Authors:** Iina Tolonen, Aino Saarinen, Liisa Keltikangas-Järvinen, Virva Siira, Mika Kähönen, Mirka Hintsanen

**Affiliations:** ^1^Unit of Psychology, Faculty of Education, University of Oulu, Oulu, Finland; ^2^Department of Psychology and Logopedics, Faculty of Medicine, University of Helsinki, Helsinki, Finland; ^3^Department of Clinical Physiology, Tampere University Hospital and Faculty of Medicine and Health Technology, Tampere University, Tampere, Finland

**Keywords:** compassion, personality, job demand control, effort-reward imbalance, longitudinal

## Abstract

Dispositional compassion has been shown to predict higher well-being and to be associated with lower perceived stress and higher social support. Thus, compassion may be a potential individual factor protecting from job strain. The current study examines (i) whether dispositional compassion predicts job strain and effort-reward imbalance (ERI) or does the predictive relationship run from job strain and ERI to dispositional compassion and (ii) the effect of dispositional compassion on the developmental trajectory of job strain and ERI over a 11-year follow-up. We used data from the Young Finns study (*n*=723) between 2001 and 2012. The direction of the predictive relationships was analyzed with cross-lagged panel models. Compassion’s effect on the trajectories of job strain, ERI, and their components was examined with multilevel models. First, the cross-lagged panel models demonstrated there was no evidence for the predictive pathways between compassion and job strain or its components. However, the predictive pathways from high dispositional compassion to low ERI and high rewards had better fit to the data than the predictive pathways in the opposite direction. In addition, multilevel models showed that high compassion predicted various job characteristics from early adulthood to middle age (lower job strain and higher job control as well as lower ERI and higher reward). Compassion did not predict job demand/effort. The findings were obtained independently of age, gender, and socioeconomic factors in childhood and adulthood. These findings indicate that compassion may be beneficial in work context. Further, compassion might be useful in the management or prevention of some aspects of strain. Our study provides new insight about the role of compassion in work life.

## Introduction

Dispositional compassion can be defined as a disposition to experience concern for others’ suffering and a subsequent desire to alleviate the perceived suffering (Goetz et al., 2010). For the past 20years, an increasing body of literature has emerged on the benefits of compassion. For instance, compassion has been shown to increase prosocial behaviors while also being associated with lower anxiety and greater positive affect in the workplace (Lilius et al., 2008; Dutton et al., 2014; Luberto et al., 2018). Recently, compassion has been found to be beneficial for the compassionate individuals themselves, for example, in reducing depressive and anxiety symptoms (Kirby et al., 2017; Saarinen et al., 2019). Yet, in the current state, the literature provides no knowledge on the relationship between dispositional compassion and job strain, a common source of psychological distress (Rigó et al., 2020). Hence, the aim of the present study is to examine the association of compassion with strain indicated by the demand-control model and effort-reward imbalance (ERI) model. We will examine this from two perspectives: whether dispositional compassion predicts strain or vice versa and whether dispositional compassion can predict strain over a 11-year follow-up.

As much as a quarter of people in Europe have reported experiencing job strain and the prevalence appears to be increasing (EU-OSHA, 2009; Eurofound and EU-OSHA, 2014; Rigó et al., 2020). Job strain is associated with a variety of health risks and indicators of ill-being, including lower quality of life (Pekkarinen et al., 2004), psychopathology (Mahan et al., 2010; Stansfeld et al., 2012), and an increased risk for a cardiovascular disease (Kuper and Marmot, 2003). The consequences of job strain also include extensive economic costs. Companies may experience financial losses, as job strain is associated with increased absenteeism and turnover intentions (Wang et al., 2014; Liu et al., 2019). Further, it has been estimated that, for instance in the United States, job strain annually costs $180 billion through the healthcare system (Goh et al., 2016).

Job characteristics, such as high work intensity, lack of autonomy, and job uncertainty, are some of the antecedents of job strain (Karasek, 1979; Siegrist, 1996; Demerouti et al., 2001). Job demand-control model (Karasek, 1979) and ERI model (Siegrist, 1996) are two of the prominent models in job strain research. The job demand-control model postulates that together high job demand and low job control are a source of job strain. If prolonged or frequent, job strain can transform into physiological and psychological arousal and finally into pervasive distress, if not addressed appropriately (Karasek, 1979). While job demand refers to the amount of time, energy, and effort put into job tasks, job control stands for one’s freedom to apply one’s professional skills, to control work pace, and to practice autonomy (Karasek, 1979). On the other hand, ERI model proposes that high efforts in relation to disproportionally low rewards cause stress at work (Siegrist, 1996). The imbalance is stressful as it goes against the core assumption of reciprocity and equal exchange of social and material commodities (Siegrist et al., 2004). As an employee confronts a variety of challenges, obligations, and situations requiring coping skills at work (i.e., effort), the expectation is to be compensated in the form of, for example, increased professional esteem, promotion opportunities, or job security (i.e., reward; Siegrist et al., 2004).

One of the major milestones in adulthood is inclusion in the work life, whereas in some degree, job strain is inevitable part of work life. Individual differences are determining factors in the experience of stress and strain, as they influence perception, interpretation, and coping mechanisms (e.g., Lazarus and Folkman, 1984). Further, the occupational stress research has highlighted the importance of personal resources in dealing with job strain (Xanthopoulou et al., 2007). Indeed, in a recent study, it was found that the use maladaptive coping strategies increased as job strain increased, an indicative of the need for personal resources aiding in self-regulation (Bakker and de Vries, 2021).

Previously, it has been shown that dispositional traits like high neuroticism, high negative emotionality, and low sociability are associated with higher job strain and ERI (Hintsanen et al., 2011; Törnroos et al., 2012, 2013). On the other hand, dispositional mindfulness has been shown to protect against job strain (Bostock et al., 2019). Another protective disposition may be high compassion. To demonstrate, it has been suggested that training compassion can decrease perceived stress (Matos et al., 2017; Brito-Pons et al., 2018), alleviate burnout symptoms, and increase resilience and job satisfaction (Kok et al., 2013; Scarlet et al., 2017; Orellana-Rios et al., 2018). However, in the current state of the literature, it is unknown if compassion serves as a protective factor against job strain or if, instead, job strain reduces compassion. Consequently, longitudinal research is also absent concerning compassion and job strain.

However, there are several reasons why dispositional compassion may predict job strain, rather than vice versa. First, compassion may be strain-buffering through the heightened affiliative experiences, which have repeatedly been found to predict lesser stress (for a review, see Taylor, 2011). Compassion has been tightly linked with higher social support and connectedness (Crocker and Canevello, 2008), shown to predict higher social support in a longitudinal manner (Saarinen et al., 2020b), and to moderate the effectiveness of social support in stressful situations (Cosley et al., 2010). Second, the highly compassionate individuals may perceive and cope with job strain in a specific manner. Already in compassion’s definition “desire to alleviate the perceived suffering,” it is implicated that compassion entails distress tolerance (Goetz et al., 2010). However, it is noteworthy that such emotional engagement may also predispose to job strain through increased demands. Nevertheless, past compassion research has shown that compassionate individuals have higher acceptance when faced with psychosocial stressors (Jazaieri et al., 2018) and compassionate individuals tend to create an upward spiral of positive emotions in stressful situations (Kok et al., 2013; Weng et al., 2013). Third, greater physical well-being may serve as protective element against stress, as compassion is related to greater physical well-being. For instance, compassion has been found to be linked with an increase in the activity in the parasympathetic nervous system (Kim et al., 2020), whereas high ERI has been associated with a decrease in such activity (Hintsanen et al., 2007). Further, compassion has been linked with physical well-being markers, such as lower blood pressure (Saarinen et al., 2020a), while job strain is known to cause hypertension (Kuper and Marmot, 2003).

However, the predictive relationship may also operate in the opposite direction: lesser job strain may help foster compassion. A work environment including supportive colleagues, professional progression, and autonomy (i.e., less job strain) may encourage compassion as they are suggested to build up resources (Xanthopoulou et al., 2007). The already cooperative work environments may inspire the individual to be more compassionate, as prosocial behaviors are found to be contagious while also associated with lower stress (Fowler and Christakis, 2010; Raposa et al., 2016). In addition, it is possible that the association between high compassion and low job strain may emerge due to collective compassion among employees, rather than due to intrapersonal compassion (Lilius et al., 2008).

Hence, as the temporal and longitudinal relationships of compassion and job strain have not yet been studied, the aims of the current study are (i) to investigate the predictive relationships of dispositional compassion with job strain and ERI and their components (i.e., does dispositional compassion predict job strain and ERI, or vice versa) and (ii) to investigate the longitudinal effect of dispositional compassion on the developmental trajectory of job strain and ERI and their components from early adulthood to middle age. Compassion has been linked with greater psychological well-being as well as mechanisms supporting better stress management, such as good emotion regulation and social support (Cosley et al., 2010; Jazaieri et al., 2014; Saarinen et al., 2020b). Hence, we hypothesize that the direction of the relationship proceeds from high compassion to lower job strain and ERI, and high compassion predicts lower job strain and ERI in a longitudinal manner.

## Materials and Methods

### Participants

We used population-based data from the prospective Young Finns study that investigates the risk factors and determinants of cardiovascular disease. The participants were randomly selected from the national population register and at the time lived in five Finnish medical school cities or their rural surroundings (Helsinki, Turku, Tampere, Kuopio, Oulu). The study includes participants from six age cohorts (born in 1962, 1965, 1968, 1971, 1974, and 1977). The baseline assessment was conducted in 1980 when the participants were aged from 3 to 18years (*N*=3,596). Eight follow-ups have been carried out since then. The current study used the follow-ups of 2001 and 2012 (when participants were aged from 35 to 50) for dispositional compassion, and the follow-ups of 2001, 2007, and 2012 for job strain and ERI. Childhood socioeconomic factors were assessed in 1980 and participants’ adulthood socioeconomic factors in 2011. The study received ethical approval from all the Ethical Committees of the Finnish Medical Schools involved in the study. Current research was conducted in accordance with the Declaration of Helsinki. Written informed consent was obtained from all the participants, or from their parents in case participants were under 18years old.

In the current study, we included only participants who were working full time during the follow-ups (2001, 2007, and 2011; *n*=918). Of these, we included only participants who had full data on the covariates (age, gender, childhood, and adulthood socioeconomic factors; *n*=792). Lastly, we included in the analyses only participants who had data available on compassion at least in one of the follow-ups (*n*=724) and data on job strain and ERI in at least one of the follow-ups (*n*=723). Hence, the final number of participants in our analyses was 723.

### Measures

#### Dispositional Compassion

Dispositional compassion for others was assessed with a subscale from Cloninger’s Temperament and Character Inventory (“compassion vs. revengefulness”; Cloninger et al., 1994). The scale includes 10 items, including “I hate to see anyone suffer” and “It gives me pleasure to see my enemies suffer” (a reversed item). Participants provided their responses using a 5-point Likert scale, ranging from 1 (“Completely disagree”) to 5 (“Completely agree”). The internal consistency of the scale in the current study was high (Cronbach’s *α*=0.86 in 2001; Cronbach’s *α*=0.85 in 2012). Further, the test-retest correlation between 2001 and 2012 was high (*r*=0.687, *p*<0.001). The construct validity and reliability of the compassion scale have been found to be good in the past and are described in more detail elsewhere (Saarinen et al., 2020b). The mean score of the scale was calculated for all the participants who had responded to at least 50% of the items.

#### Job Strain

Job strain was measured by using three items from the Occupational Stress Questionnaire (OSQ) developed at the Finnish Institute of Occupational Health (Elo et al., 1992) and nine items from the Job Content Questionnaire (JCQ; Karasek, 1985). The job demand items were comparable to the Karasek’s Job Content Questionnaire and were the following: (1) “Do you have to hurry to get your work done?,” (2) “Does your work have phases that are too difficult?,” and (3) “Is your work mentally strenuous?.” Job control was assessed with nine items from the JCQ. The items of job demand and job control were responded on a 5-point Likert scale ranging from 1 (“Completely disagree”) to 5 (“Completely agree”).

Job demand and control were measured in 2001 (job demand Cronbach’s *α*=0.56; job control *α*=0.86), 2007 (job demand *α*=0.60; job control *α*=0.86), and 2012 (job demand *α*=0.61; job control *α*=0.84). The inter-item correlation values for job demand were 0.23 (2001), 0.24 (2007), and 0.23 (2012), while the corrected item-total correlations were 0.68–0.78 (2001), 0.69–0.78 (2007), and 0.70–0.78 (2012). The inter-item correlation values for job control were 0.42 (2001), 0.38 (2007), and 0.34 (2012) and the corrected item-total correlations were 0.51–0.75 (2001), 0.52–0.79 (2007), and 0.48–0.79 (2012). The mean scores of job demand and control were calculated for those participants who had at least 50% of the data available on each of these scales.

The total job strain was calculated as the subtraction of the mean score of job control from the mean score of job demand (job strain=job demand-job control). The calculation captures the potential additive effects of job demand and job control (i.e., job strain increases as job demand increases and job control decreases). Previous research has recommended to use the continuous measure and to equally weigh the contribution of job demand and job control for job strain (Landsbergis et al., 1994; MacCallum et al., 2002).

#### Effort-Reward Imbalance

The effort items were taken from the OSQ (Elo et al., 1992) and were the same as the job demand items. The three reward items were also from the OSQ (Elo et al., 1992) and were the following: (1) “Do you get help and support from your superior if needed?” (“1=Very little”; “5=Very much”), (2) “How do your co-workers get along with each other at the workplace?” (Their relationship is: “1=Bad, tense, resentful, etc.”; “5=Very good”), and (3) “How satisfied are you with your current employment?” (“1=Very unsatisfied”; “5=Very satisfied”). The reward items came from 2001 (*α*=0.58), 2007 (*α*=0.52), and 2012 (*α*=0.54). The mean scores of reward were calculated for those participants who had at least 50% of the data available. Inter-item correlations for reward were 0.25 (2001), 0.26 (2007), and 0.28 (2012). The corrected item-total correlations were 0.71–0.79 (2001), 0.67–0.75 (2007), and 0.69–0.77 (2012) for reward.

The ERI was calculated by dividing the mean score of effort by the mean score of reward (ERI=effort/reward) as recommended in past research (Siegrist et al., 2004). The currently employed ERI items have been used in previous research (Hintsanen et al., 2007, 2011). Further, the currently used proxy effort and reward correlate with the original effort and reward (*r*=0.631, *r*=0.587, *p*<0.001, respectively; Hintsanen et al., 2011).

#### Covariates

Childhood socioeconomic status was assessed by parents’ self-reports, including parental occupational status, parental educational level, and family income in 1980. Participants’ adulthood socioeconomic status was measured by participants’ self-reported occupational status, educational level, and level of income in 2011. The occupational status of the participants and their parents was coded as “1=Manual,” “2=Lower grade non-manual,” or “3=Upper grade non-manual.” Educational level of the participants and their parents included three categories (“1=Comprehensive school,” “2=High school or vocational school,” and “3=Academic level, i.e., university or college”). If the parental educational level or occupational status differed between the parents, the higher level or status was selected. Level of family income (1980) was reported with an 8-point scale (“1=Less than 15000 Finnish marks per year”; “8=More than 100000 Finnish marks per year”). Participants’ adulthood income in 2011 was reported with a 13-point scale (“1=Less than 5000€”; “13=More than 60000€”). Each socioeconomic factor was entered as a separate variable in the analyses. Educational level and occupational status were treated as categorical variables and income as a continuous variable.

### Statistical Analyses

The data were analyzed with the statistical software Stata SE 16.1. Attrition analysis was conducted by comparing the included (*n*=723) and excluded (*n*=2873) participants regarding the study variables using independent t-tests and chi-square independence tests.

First, cross-lagged panel models were used to examine the predictive relationship of compassion with job strain and ERI as well as their components in 2001 and 2012. Four models were estimated as: (1) no predictive pathways between compassion and job characteristics (i.e., only autoregressive paths), (2) a predictive regression path from compassion to job characteristics, (3) a predictive regression path from job characteristics to compassion (i.e., in the opposite direction), and (4) bidirectional predictive regression paths in both directions between compassion and job characteristics. All the models were adjusted for age, gender, and socioeconomic factors in childhood and adulthood. In all the models, we included covariances between compassion and job characteristics at each measurement year. The Root Mean Square Error of Approximation (RMSEA), Comparative Fit Index (CFI), Bayesian Information Criterion (BIC) scores, and *χ*2 test difference value were used to evaluate the statistical fit of the models. Past research has suggested that values of RMSEA that are below 0.06, the values of CFI above 0.95, and lower scores of BIC and *χ*2 indicate a good statistical fit of the model (Hu and Bentler, 1999; Schreiber et al., 2006).

Second, we investigated the longitudinal relationships of compassion (measured in 2001) with job strain and ERI as well as their components (measured in 2001, 2007, and 2012). To do this, we used multilevel models with maximum likelihood estimation. The developmental curve of job characteristics was predicted by compassion in 2001. Intercept, compassion in 2001, age, age squared, gender, and socioeconomic factors in childhood and adulthood were set as fixed effects (i.e., the constant variables across individuals estimated with maximum likelihood). Random effects (i.e., the source of random variation) included within-individual variation over the follow-up (i.e., residual variance). Participants’ age ranged between 24 and 50 years over the follow-up of job strain, ERI, and their components. Participants’ age was centered to the age of the youngest age cohort in the first measurement year of the outcome variable (2001; i.e., 24years). This was done to reduce potential multicollinearity.

## Results

Descriptive statistics are shown in Table 1. Attrition analyses showed that there were no differences in compassion (2001) between included and excluded participants (*p*>0.05). Furthermore, there were no differences in gender, job demand (2001), adulthood occupational status (2011), family income in childhood (1980), parental occupational status (1980), or parental education level (1980) between included and excluded participants (*p*>0.05). Included participants were older compared to excluded participants (*M*=32.7 vs. *M*=31.1, *p*<0.001). Included participants also had lower job strain (2001; *M*=−0.98 vs. *M*=−0.89, *p*<0.05), higher job control (2001; *M*=3.86 vs. *M*=3.74, *p*<0.001), lower ERI (2001; *M*=0.78 vs. *M*=0.82, *p*<0.01), and higher reward (2001; *M*=3.86 vs. *M*=3.70, *p*<0.001) compared to the excluded participants. Included participants had higher income (2011; *M*=8.35 vs. *M*=6.79, *p*<0.001) and were more likely to have an academic education (2011; 40.4% vs. 25.9%, *p*<0.001) than excluded participants.

**Table 1:** The means, standard deviations (*SD*), and frequencies of the study variables.

Variable (Measurement year)	Mean/Frequency(%)	*SD*	Range
Compassion (2001)	3.694	0.617	1–5
Job strain^a^ (2001)	−0.978	0.874	−3.333–2.889
Job demand^b^ (2001)	2.879	0.638	1–5
Job control (2001)	3.857	0.702	1–5
Effort-Reward Imbalance (ERI)^c^ (2001)	0.779	0.275	0.230–3.333
Reward (2001)	3.864	0.666	1–5
Gender (Female)	344 (47.6%)		
Age (2001)	32.7	4.9	24–39
Family income (1980)	4.881	1.884	1–8
Participants’ level of income (2011)	8.349	2.728	1–13
**Parental educational level (1980)**
Comprehensive school	250 (34.6%)		
High school or occupational school	305 (42.2%)		
Academic level (university or college)	168 (23.2%)		
**Parental occupational status (1980)**
Manual	289 (40.0%)		
Lower grade non-manual	313 (43.3%)		
Upper grade non-manual	121 (16.7%)		
**Participants’ educational level (2011)**
Comprehensive school	14 (1.9%)		
High school or occupational school	417 (57.7%)		
Academic level (university or college)	292 (40.4%)		
**Participants’ occupational status (2011)**
Manual	360 (49.8%)		
Lower grade non-manual	148 (20.5%)		
Upper grade non-manual	215 (29.7%)		

a Job strain=job demand-job control.

b Job demand and effort were measured with the same items.

c ERI=effort/reward.

### The Predictive Pathways of Compassion With Job Strain and ERI

Table 2 presents the results of cross-lagged panel models for the predictive relationships of compassion with job strain and its components job demand and job control. All the models were adjusted for age, gender, and socioeconomic factors in childhood and adulthood. First, the results of compassion and job strain indicated that all the models had good statistical fit indices (RMSEA≤0.039, CFI≥0.977). There was no evidence for statistical fit differences between Models 1, 2, 3, and 4 in the *χ*^2^ difference test (*p*>0.05). Altogether, the results indicated that there were no predictive relationships between compassion and job strain. Second, we conducted cross-lagged panel models using the components of job strain: job demand and job control (instead of job strain). When examining the relationships of compassion with job demand and job control, the models had good and adequate statistical fits, respectively. Further, there was no evidence that the models differed from one another in the *χ*^2^ difference tests (*p*>0.05). Hence, the results indicated that there were no predictive relationships of compassion with job demand or job control.

**Table 2:** The goodness-of-fit statistics for the cross-lagged panel models on the predictive relationships of compassion with job strain and its components (adjusted for age, gender, and socioeconomic factors in childhood and adulthood).

							Model comparisons		
	*χ*^2^ value	*df*	*p*	RMSEA	CFI	BIC	*χ*^2^ difference test	*df*	*p*
**Job strain**									
Model 1	29.799	18	0.039	0.035	0.979	16466.617			
Model 2	28.801	17	0.036	0.036	0.979	16471.906	*χ*^2^(2 vs. 1) = 1.00	1	0.318
Model 3	29.770	17	0.028	0.037	0.977	16472.875	*χ*^2^(3 vs. 1) = 0.03	1	0.886
Model 4	28.758	16	0.026	0.039	0.977	16478.149	*χ*^2^(4 vs. 1) = 1.04	2	0.594
**Job demand**									
Model 1	39.293	18	0.003	0.047	0.959	15965.732			
Model 2	38.793	17	0.002	0.049	0.958	15971.522	*χ*^2^(2 vs. 1) = 0.50	1	0.479
Model 3	39.231	17	0.002	0.049	0.957	15971.960	*χ*^2^(3 vs. 1) = 0.06	1	0.803
Model 4	38.732	16	0.001	0.051	0.956	15977.751	*χ*^2^(4 vs. 1) = 0.56	2	0.755
**Job control**									
Model 1	69.374	18	<0.001	0.073	0.930	15791.427			
Model 2	66.704	17	<0.001	0.074	0.933	15795.042	*χ*^2^(2 vs. 1) = 2.67	1	0.102
Model 3	69.373	17	<0.001	0.076	0.929	15797.711	*χ*^2^(3 vs. 1) = 0.00	1	0.971
Model 4	66.703	16	<0.001	0.077	0.931	15801.327	*χ*^2^(4 vs. 1) = 2.67	2	0.263

Model 1: No cross-lagged predictive paths. Model 2: Predictive path from compassion to job characteristics. Model 3: Predictive path from job characteristics to compassion. Model 4: Bidirectional predictive paths.

**Figure 1:**
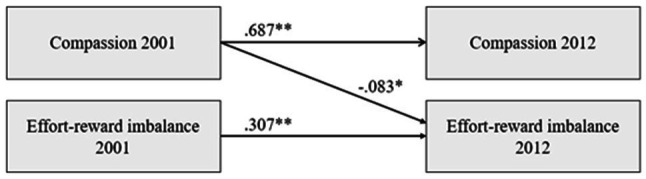
The illustration of the predictive pathways from compassion to ERI. Covariates (age, gender, and socioeconomic factors in childhood and adulthood) and the covariances between the study variables at each measurement year have been omitted from the figure for clarity. ^*^p<0.05, ^**^p<0.001.

Table 3 presents the results of cross-lagged panel models for the predictive relationships of compassion with ERI and its component reward. All the models were adjusted for age, gender, and socioeconomic factors in childhood and adulthood. Regarding the predictive relationships between compassion and ERI, it was found that all the models had good statistical fit indices (RMSEA≤0.035, CFI≥0.976). The *χ*^2^ difference tests, CFI values, RMSEA values, and BIC-values showed that Model 2 (a predictive relationship from compassion to ERI) was better than Model 1 (no predictive pathways; *p*<0.05 in the *χ*^2^ difference test) and Model 3 (predictive pathway from ERI to compassion). Model 4 (bidirectional predictive pathways) and Model 2 did not differ from one another in the *χ*^2^ difference test (*p*>0.05 in the *χ*^2^ difference test). Taken together, the predictive pathways are more likely to run from compassion to ERI than in the opposite direction. Depiction of the pathway is presented in Figure 1.

**Table 3:** The goodness-of-fit statistics for the cross-lagged panel models on the predictive relationships of compassion with ERI and its component (adjusted for age, gender, and socioeconomic factors in childhood and adulthood).

							Model comparisons		
	*χ*^2^ value	*df*	*p*	RMSEA	CFI	BIC	*χ*^2^ difference test	*df*	*p*
**ERI**									
Model 1	28.356	18	0.057	0.033	0.977	14238.794			
Model 2	24.359	17	0.110	0.028	0.984	14241.079	*χ*^2^(2 vs. 1) = 4.00	1	0.046
Model 3	28.171	17	0.043	0.035	0.976	14244.892	*χ*^2^(3 vs. 1) = 0.18	1	0.668
Model 4	24.214	16	0.085	0.031	0.982	14247.217	*χ*^2^(4 vs. 1) = 4.14	2	0.126
							*χ*^2^(4 vs. 2) = 0.14	1	0.704
**Reward**									
Model 1	37.327	18	0.005	0.045	0.959	16063.770			
Model 2	24.077	17	0.117	0.028	0.985	16056.802	*χ*^2^(2 vs. 1) = 13.25	1	<0.001
Model 3	36.755	17	0.004	0.047	0.959	16060.480	*χ*^2^(3 vs. 1) = 0.57	1	0.450
Model 4	23.685	16	0.097	0.030	0.984	16062.692	*χ*^2^(4 vs. 1) = 13.64	2	0.001
							*χ*^2^(4 vs. 2) = 0.39	1	0.531

Model 1: No cross-lagged predictive paths. Model 2: Predictive path from compassion to job characteristics. Model 3: Predictive path from job characteristics to compassion. Model 4: Bidirectional predictive paths.

**Figure 2:**
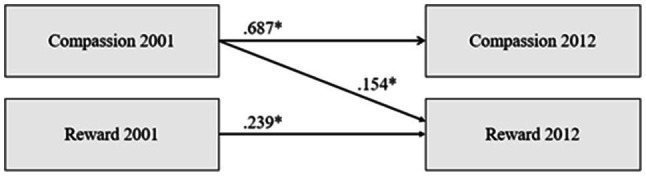
The illustration of the predictive pathways from compassion to reward (a component of ERI). Covariates (age, gender, and socioeconomic factors in childhood and adulthood) and the covariances between the study variables at each measurement year have been omitted from the figure for clarity. ^*^p<0.001.

We also investigated the predictive relationship of compassion with reward (a component of ERI). The goodness-of-fit indices showed that all models had good statistical fit indices (RMSEA≤0.047, CFI≥0.959). Further, Model 2 (a predictive relationship from compassion to reward) had better statistical fit than Model 1 (no predictive pathways) in the *χ*^2^ difference test (*p*<0.001) or Model 3 (a predictive pathway from reward to compassion). Model 4 (bidirectional predictive paths) and Model 2 did not differ from one another in the *χ*^2^ difference test (*p*>0.05). Taken together, the results indicated that the predictive pathways are more likely to run from compassion to reward than in the opposite direction. Depiction of the pathway is presented in Figure 2.

### The Longitudinal Relationship of Compassion With Job Strain and ERI

**Figure 3:**
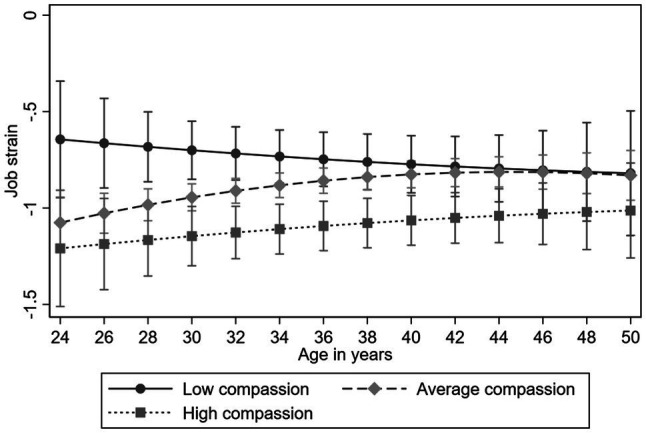
Model-predicted values with 95% confidence intervals of job strain over age separately for participants with low (−1*SD*), average, and high (+1*SD*) levels of compassion (adjusted for gender and childhood and adulthood socioeconomic factors).

**Figure 4:**
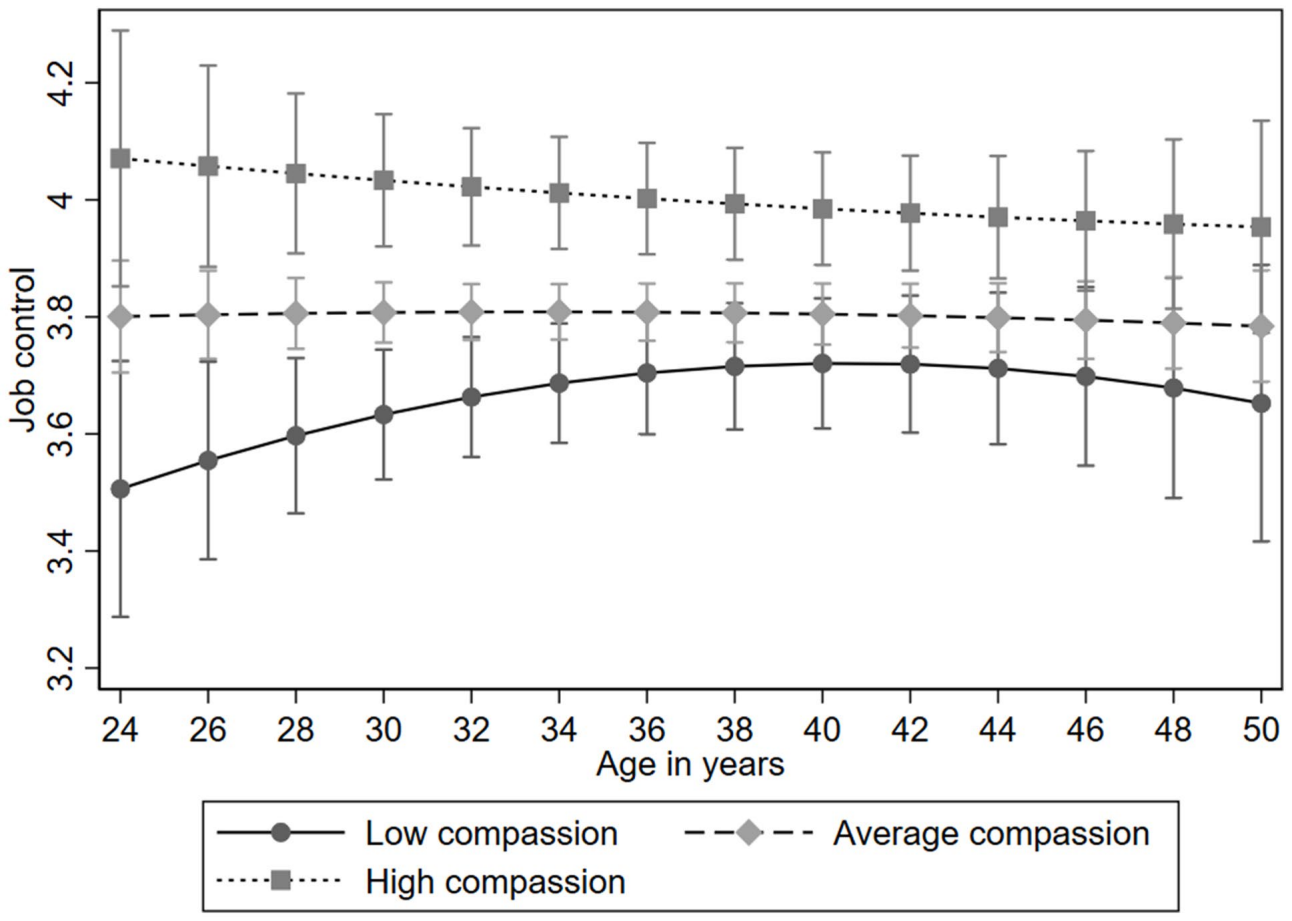
Model-predicted values with 95% confidence intervals of job control (component of job strain) over age separately for participants with low (−1*SD*), average, and high (+1*SD*) levels of compassion (adjusted for gender, and socioeconomic factors in childhood and adulthood).

We examined the longitudinal association of compassion on the trajectories of job strain and its components. The results of the multilevel models are presented in Table 4. All the findings were adjusted for gender and participants’ childhood and adulthood socioeconomic factors. The results indicated that compassion predicted lower job strain (*B*=−0.292, *p*<0.01). High compassion also predicted higher job control (*B*=0.282, *p*<0.001). There was no evidence for the compassion-age interaction in the analyses of job strain or its components (*p*>0.05), indicating the effect was evident over the 11-year follow-up (see Figures 3, 4, for job strain and job control, respectively). Compassion did not predict job demand (*p*>0.05).

**Table 4:** Results of multilevel models with a longitudinal design. Estimates (B) with 95% confidence intervals (CI) of compassion and age, when predicting job strain and its components (adjusted for gender and socioeconomic factors in childhood and adulthood).

	Job strain		Job demand		Job control	
	*B*	95% CI	*B*	95% CI	*B*	95% CI
**Fixed effects**						
Intercept	0.589	−0.214; 1.391	2.566^***^	1.960; 3.171	1.974^***^	1.374; 2.575
Age	−0.026	−0.138; 0.087	0.042	−0.043; 0.127	0.068	−0.016; 0.153
Compassion×Age	0.010	−0.020; 0.040	−0.006	−0.028; 0.017	−0.016	−0.038; 0.007
Compassion×Age×Age	−0.0002	−0.001; 0.001	0.0003	−0.001; 0.001	0.001	−0.0003; 0.001
Compassion	−0.292^**^	−0.481; −0.104	−0.012	−0.155; 0.130	0.282^***^	0.140; 0.423
**Random effects**						
Residual variance	0.651^*^	0.624; 0.679	0.492^*^	0.472; 0.512	0.490^*^	0.469; 0.511

*n*=723;

* *p*<0.05,

** *p*<0.01,

*** *p*<0.001.

**Figure 5:**
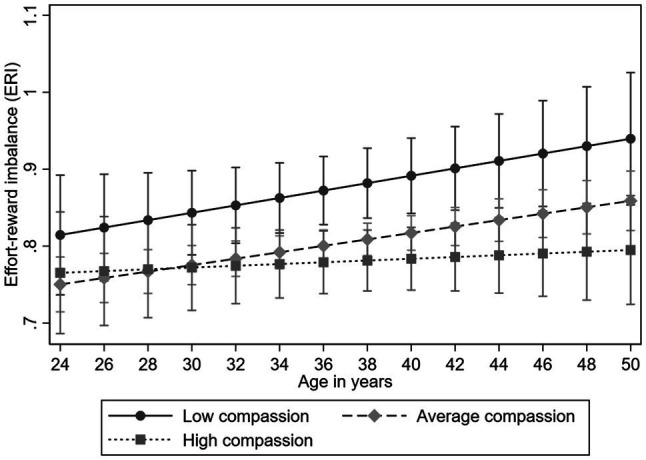
Model-predicted values with 95% confidence intervals of ERI over age separately for participants with low (−1*SD*), average, and high (+1*SD*) levels of compassion (adjusted for gender, and socioeconomic factors in childhood and adulthood).

**Figure 6:**
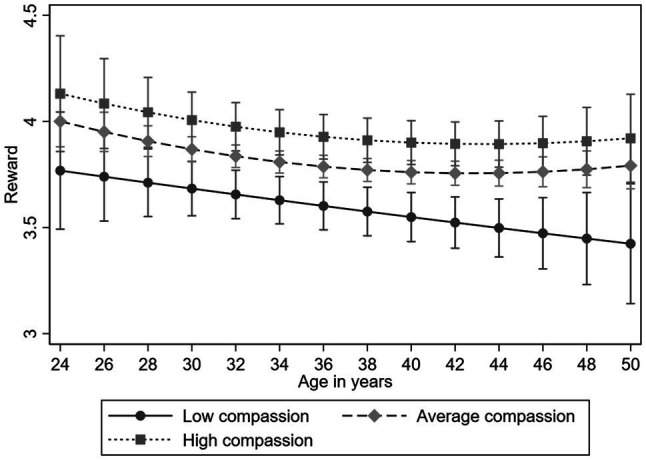
Model-predicted values with 95% confidence intervals of reward (component of ERI) over age separately for participants with low (−1*SD*), average, and high (+1*SD*) levels of compassion (adjusted for gender, and socioeconomic factors in childhood and adulthood).

Lastly, we examined the longitudinal association of compassion on the trajectories of ERI and its component reward. The results of the multilevel models are presented in Table 5. All the findings were adjusted for gender and participants’ childhood and adulthood socioeconomic factors. High compassion predicted lower ERI (*B*=−0.056, *p*<0.05). In addition, compassion predicted higher rewards (*B*=0.237, *p*<0.01) over the follow-up. There was no evidence for the compassion-age interaction in either of these analyses (*p*>0.05), indicating that the effect of compassion on ERI and reward was evident between the years 2001 and 2012 (see Figures 5, 6, for ERI and reward, respectively).

**Table 5:** Results of multilevel models with longitudinal design. Estimates (B) with 95% confidence intervals (CI) of compassion and age, when predicting ERI and its component reward (adjusted for gender and socioeconomic factors in childhood and adulthood).

	ERI		Reward	
	*B*	95% CI	*B*	95% CI
**Fixed effects**				
Intercept	1.004^*^	0.787; 1.220	2.626^***^	1.926; 3.326
Age	0.007	−0.006; −0.010	0.006	−0.094; 0.105
Compassion×Age	−0.001	−0.004; 0.003	−0.008	−0.035; 0.018
Compassion×Age×Age^d^			0.0003	−0.001; 0.001
Compassion	−0.056^*^	−0.102; −0.010	0.237^**^	0.070; 0.413
**Random effects**				
Residual variance	0.236^*^	0.226; 0.246	0.582^*^	0.559; 0.606

d When predicting ERI, there was no evidence for the compassion×age squared interaction and thus, excluded from the model, *n*=723.

* *p*<0.05,

** *p*<0.01,

*** *p*<0.001.

## Discussion

The current study is the first to investigate the association between dispositional compassion, job strain, and ERI. Our results with a population-based sample and a 11-year follow-up design showed that high dispositional compassion predicted lower job strain and ERI. More specifically, the results from cross-lagged panel models indicated that predictive pathways from high compassion to lower ERI and higher reward had better fit to the data than the predictive pathways in the opposite direction. We did not find evidence for the predictive relationships between dispositional compassion and job strain, job demand/effort, or job control. Second, multilevel models showed that dispositional compassion predicted the developmental trajectory of several job characteristics. High compassion predicted lower job strain and higher job control from early adulthood into middle age. Similarly, we found that high compassion predicted lower ERI and higher reward throughout the follow-up (2001–2012). Compassion was not related to job demand/effort. Age, gender, and socioeconomic factors in childhood and adulthood were taken into account in all the analyses.

Past research has suggested that compassion training can decrease perceived stress (Matos et al., 2017; Brito-Pons et al., 2018) as well as improve burnout symptoms and job satisfaction (Scarlet et al., 2017; Orellana-Rios et al., 2018). The current results align with these findings and further contribute to the existing literature by showing that dispositional compassion is a relevant, individual factor in job strain, ERI, and their components. Furthermore, our results indicated that high compassion may function as a protective factor against ERI, perhaps, through high reward.

One form of reward is socio-emotional reward, such as social support, and the measured rewards strongly reflect such type of a reward. The role socio-emotional reward in buffering stress is well established (Siegrist et al., 2004; for a review, see Taylor, 2011). Furthermore, past research has found that compassion does not only predict higher social support but also moderates the effectiveness of social support (Cosley et al., 2010; Saarinen et al., 2020b), whereas the core of ERI is within the assumption of social reciprocity (Siegrist, 1996). Highly compassionate individuals may be able to mobilize more social support and/or are more proficient in capitalizing it, resulting in both psychological and physiological stress relief. Compassionate individuals may perceive greater baseline social connectedness, are more capable in creating supportive environments for themselves, or have a lower threshold to seek social support. Thus, compassion’s protective effect against stress may emanate from higher, real or perceived, social connectedness. Indeed, the interrelationship of compassion, social connectedness, and biological stress-relieving mechanisms, such as the dampening of the HPA-axis reactivity, has received empirical evidence (Cosley et al., 2010; Abelson et al., 2014; Erickson et al., 2017).

In addition, we also found that high compassion predicted higher reward over the period of 11years. A relatively recent review highlighted that compassion is connected to and, more importantly, may be partly responsible for the activation of the neural reward circuitry (Klimecki, 2015). The act of imagining being compassionate (i.e., compassion meditation) has been shown to activate brain regions associated with reward as well as to reduce stress and cortisol levels (Abelson et al., 2014; Klimecki et al., 2014; Brito-Pons et al., 2018). The activation of reward pathways induces a variety of outcomes including dopamine production and feelings of pleasure and positive affect, leading to endogenous stress relief and resilience (for a review, see Dutcher and Creswell, 2018). Past studies have found that compassionate individuals do not attempt to suppress their negative affect, but rather elevate their positive affect and show greater acceptance in a stressful situation (Kok et al., 2013; Weng et al., 2013; Engen and Singer, 2015; Jazaieri et al., 2018). Altogether, the findings of the past research indicate that compassion may be associated with a specific pattern of emotion regulation strategy, which may aid in the management of and buffering against stress and strain (Abelson et al., 2014; Jazaieri et al., 2014).

The broaden-and-build theory corroborates with the above-mentioned and current findings, as positive emotions have been found to broaden cognitive repertoire, build up resources, and thus, support resilience (Fredrickson, 2001; Fredrickson et al., 2008). In contrast, negative emotions appear to narrow one’s attentional capacity and motivate to escape, potentially leading to maladaptive coping strategies (Fredrickson, 2001; Bakker and de Vries, 2021). Consequently, maladaptive coping strategies increase job strain while negative emotionality has been found to be associated with lower job control and lower rewards (Hintsanen et al., 2011; Bakker and de Vries, 2021). In contrast, we found the opposite results with compassion in the current study. Thus, compassion’s positive bias may support strain-buffering and emotion regulation skills in the compassionate individuals (e.g., Jazaieri et al., 2014; Engen and Singer, 2015).

We found somewhat discrepant findings concerning the associations of compassion with job strain as compared to those with ERI. However, past research has suggested that job strain and ERI should not be perceived as competing models, but as complementing ones as they appear to contribute differently to the literature (de Jonge et al., 2000; Calnan et al., 2004). For instance, the current results showed that job strain appeared to attenuate among the least compassionate individuals from young adulthood to middle age. On the other hand, the least compassionate individuals showed increasing amounts of ERI over the 11years. Depending on the occupational characteristics, psychosocial stressors may also vary greatly, which may be captured by the apparent discrepant results of job strain and ERI. Furthermore, over time, people may accumulate their professional skills, find efficient coping strategies, and build other means to deal with job strain and ERI. Thus, compassion is likely to be just one of the many contributors to stress management.

As already mentioned above, it was also found that higher compassion predicted higher job control throughout the follow-up (2001–2012). Compassion is known to have a motivational component, suggesting that compassion entails having an optimistic belief in controlling and influencing a distressing situation (Goetz et al., 2010). Moreover, it has been found that compassion predicts work engagement (De Stasio et al., 2019) while it has also been argued that training compassion increases self-efficacy through increasing calmness and reducing anxiety, although this finding needs more empirical evidence as the specific study did not employ a control group (Jazaieri et al., 2018). Nevertheless, together with the current findings, there appears to be an indication of compassionate individuals having a certain control disposition that could also translate into, for example, good job control (or at least, a perception of having opportunities at control), when facing a stressful situation at work.

It is also noteworthy that compassionate individuals may seek and/or are selected for occupations which have more favorable work environments in terms of job strain and ERI. Individual differences in motivation, cognition, and personality have been found to be factors in career decision-making process but they are also associated with varying likelihood of exposing oneself to stressors (Code and Langan-Fox, 2001; Berings et al., 2004; Mullola et al., 2018). Compassionate individuals are often characterized as kind, warm, and helpful, which can be attractive qualities from the employer’s perspective when considering, for example, teamwork skills. Moreover, compassionate individuals might gravitate toward professions and work environments which present greater amounts of rewards from the beginning. The current findings showed that the most compassionate individuals had comparatively stable perceptions of (high) reward, whereas the least compassionate individuals perceived decreasing amounts of reward over the period of 11years. While the junior employees may accept that the rewards are not as extensive in the beginning of their careers, more senior employees most likely expect increasing rewards as their careers progress. If the rewards do not increase along with the expectations, the actualized/perceived rewards do not feel adequate. Compassionate employees may obtain rewards more in quantity and quality (e.g., promotions and social support) as they have the potential to exert positive influence at work, and such behaviors are often rewarded in both official and unofficial ways. Social comparison with those employees, who receive more rewards, may further enhance the perceived imbalance between effort and reward in the less compassionate individuals.

### Limitations and Strengths

Certain limitations are present in the current study. The Cronbach’s alpha for the job demand was approximately 0.60, which is at the lower range of acceptable reliability estimates. We obtained similar Cronbach’s alpha values for reward (*α*=0.55). However, the inter-item correlation values were within a good range for both job demand and reward, thus, indicative of good internal consistency (Piedmont and Hyland, 1993). Furthermore, the corrected item-total correlations were also within good range for job demand and reward, another indicator of good internal consistency (DeVon et al., 2007). Hence, the small number of items in these scales might explain the low alpha values. Secondly, attrition analyses revealed that included participants scored lower on job strain and ERI as well as higher on job control and reward compared to the excluded participants. Taken together, this could imply that there was a bias toward lower strain in our sample. However, it should be noted that the differences between included and excluded participants were comparatively small. Finally, the current follow-up had two measurement points of compassion and three measurement points of job characteristics. The used dataset did not have compassion measurement from 2007, which would have provided more detailed information about the examined associations and their changes over time. Furthermore, while compassion is a comparatively stable personality trait (Josefsson et al., 2013), job characteristics may experience greater fluctuation over time. The fluctuations can be due to, for example, changes in one’s work tasks/responsibilities or in the event of changing jobs. Therefore, in the future studies, it would be advantageous to measure job strain and ERI in as many measurement points as possible to capture the more subtle, short-term changes.

The current study also had several, considerable strengths. The current study utilized data from a population-based 11-year prospective follow-up with three separate measurements points of job strain, ERI, and their components. This allowed the examination of predictive and longitudinal relationships from early adulthood into middle age with a relatively large sample size. By utilizing data from the general population, we were able to include a variety of occupation groups, enhancing the generalizability of our results. We also included two prominent job strain models into the study. Lastly, we controlled several confounding variables, such as age, gender, and socioeconomic factors in childhood and adulthood.

The levels of job strain appear to be increasing (EU-OSHA, 2009; Eurofound and EU-OSHA, 2014). It has been found that the rise is particularly pronounced among manual laborers and explained by increasing job demands, such as time constraints, while the perception of reward appears to be comparatively low in these positions (Rigó et al., 2020). In addition, healthcare professionals have been suggested to be at a particular risk, since estimates indicate that almost half of US physicians exhibit burnout risk (Shanafelt et al., 2019). For some time now, there has been discussion about the “cost of caring” for those in the helping professions (e.g., nurses, firefighters, police, and therapists) and a call for stress management and self-soothing techniques (Figley, 2002). The current results demonstrated evidence for compassion as a protective factor (rather than a risk) for our population-based sample. Compassion’s role in job strain, ERI, and reward can be valuable knowledge for work communities and occupational health professionals. Compassion could be, for example, included in well-being programs. As compassion can be relatively easily cultivated with interventions (Kirby et al., 2017), compassion could offer a target for intervention at workplaces in stress management and prevention. For instance, it has been suggested that 7 min of daily loving-kindness meditation (i.e., a common compassion intervention) can generate small to moderate effect sizes (Hutcherson et al., 2008). Whether compassion interventions are effective in job strain management and prevention should, however, be confirmed in future studies.

In conclusion, the current study is the first to examine dispositional compassion in relation to job strain and ERI. Thus, our study provides new insight on the role of compassion in work life. The results indicated that high compassion may buffer against ERI and increase rewards. In addition, our results showed that high compassion predicted lower job strain and higher job control over the span of 11years (from young adulthood to middle age). The current study provides new knowledge on the role of compassion in work context and may be of use when developing well-being programs at work or for occupational healthcare professionals.

## Data Availability Statement

The data analyzed in this study are subject to the following licenses/restrictions: The datasets presented in this article are not readily available because YFS is an ongoing follow-up study and the datasets are not anonymized, and the GDPR prevents public sharing of the data. Instead, pseudonymized datasets are possible to share on request and require a data sharing agreement between the parties. Requests to access these datasets should be directed to LK-J (liisa.keltikangas-jarvinen@helsinki.fi) for compassion data and to Katri Räikkönen (katri.raikkonen@helsinki.fi) or Niklas Ravaja (niklas.ravaja@helsinki.fi) for other psychological data.

## Ethics Statement

The studies involving human participants were reviewed and approved by the Ethical Committee of University of Turku (Institution name: Varsinais-Suomen sairaanhoitopiirin kuntayhtymä, Eettinen toimikunta). The patients/participants provided their written informed consent to participate in this study.

## Author Contributions

IT wrote the first draft and analyzed the data. IT, AS, and MH designed the study and collaborated on the initial interpretation of the results and writing the manuscript. AS supervised the data analyses. LK-J designed and collected the data. All authors discussed the design and the results, took part in writing the manuscript, and approved the final version.

## Funding

This study was financially supported by the Academy of Finland (MH, grant number 308676).

## Conflict of Interest

The authors declare that the research was conducted in the absence of any commercial or financial relationships that could be construed as a potential conflict of interest.

## Publisher’s Note

All claims expressed in this article are solely those of the authors and do not necessarily represent those of their affiliated organizations, or those of the publisher, the editors and the reviewers. Any product that may be evaluated in this article, or claim that may be made by its manufacturer, is not guaranteed or endorsed by the publisher.
